# Clinical Trials of Antiangiogenesis Therapy on Gastric Cancer

**DOI:** 10.4021/gr2008.11.1250

**Published:** 2008-11-20

**Authors:** Li Cun Wu, Wei Dong Zhang

**Affiliations:** a Toronto General Hospital, University Health Network (UHN), University of Toronto, Toronto, ON M5G 1L7, Canada; b Institute of Basic Medicine, Shandong Academy of Medical Sciences, Jinan 250062, China

**Keywords:** angiogenesis inhibitor, gastric cancer, clinical trial

## Abstract

Both malignant tumor growth and metastasis are dependent upon angiogenesis, a process of new blood vessel formation. Inhibition of this process by specific inhibitors might be able to control tumor growth and metastasis. Therefore, antiangiogenesis thereapy is considered a promising strategy and being studied worldwide. A wide variety of angiogenesis inhibitors have been identified and some of them are under clinical trials in the advanced patients with cancer including gastric cancer. This review summarizes the development and progress of angiogenesis inhibitors in recent decades, and discusses the future direction of antiangiogenesis research, and the potential antiangiogenic agents which are most likely to be translated into standard treatment for gastrointestinal cancer patients either alone or combined with other therapies.

## Introduction

Considerable evidences have demonstrated that the growth of malignant tumors must be dependent upon angiogenesis, the formation of new capillary blood vessels from pre-existing vasculature (1-4), and certain evidences have also showed that cancer metastasis is dependent on angiogenesis (5, 6). Once a nest of cancer cells reaches a certain size (1–2 mm in diameter), it must develop a blood supply in order to grow larger. Diffusion is no longer adequate to supply the cells with oxygen and nutrients and to take waste away (2, 4, 7).

A wide variety of angiogenesis factors have been identified by using modern biotechnology throughout the past decades (8, 9). Here are a few examples: angiopoietin-1 (10), basic fibroblast growth factor (bFGF), and vascular endothelial growth factor (VEGF) (8, 9). On the contrary, inhibition of angiogenesis can slow down tumor growth and even result in tumor regression. Meanwhile, various angiogenesis inhibitors (AIs) have been discovered and are being studied under clinical trials in the advanced patients with cancers including gastrointestinal cancer (11-13).

## Clinical trials of antiangiogenesis therapy in gastric cancer

So far, at least 21 clinical trials of antiangiogenesis therapy are being conducted in gastric cancer in 8 countries (Table 1). These trials are multi-centered and randomly controlled, most of them are in phase III clinical studies. Bevacizumab (Avastin) is widely used in these trials combined either chemotherapy or other therapies. The response rate and overall survival are encouraging, with time to disease progression improved over historical controls by 75% (13). In addition, new molecules such as Sunitinib (Sutent) and mTOR inhibitor Temsirolimus are most likely promising AIs in treating gastric cancer. Once these studies have been completed, optimization of clinically active anti-angiogenic agents will need to be further refined in order to determine where they best fit in gastric cancer, either as single agents or in combination with classical anticancer therapies. Finally, the use of these new agents may in the future encompass every aspect of cancer management, not only from palliative to curative treatment but also in the prevention of cancer.

**Table 1:** Clinical trials of antiangiogenesis therapy in gastric cancers source (from http://clinicaltrials.gov)

1.	NCT00780494: Phase II of Capecitabine, Carboplatin & Bevacizumab for Gastroesophageal Junction & Gastric Carcinoma. Stanford University and Genentech, USA
2.	NCT00394433: Docetaxel, Cisplatin, Irinotecan and Bevacizumab (TPCA) in Metastatic Esophageal and Gastric Cancer Dana-Farber Cancer Institute, Brigham and Women's Hospital, Massachusetts General Hospital, and Genentech, USA
3.	NCT00403468: Combination Chemotherapy and Bevacizumab in Treating Patients With Recurrent, Unresectable, or Metastatic Gastric Cancer, Gastroesophageal Junction Cancer, or Esophageal Cancer. Memorial Sloan-Kettering Cancer Center and National Cancer Institute (NCI), USA
4.	NCT00673673: FOLFOX With Bevacizumab in Metastatic or Unresectable Gastroesophageal and Gastric Cancer Yale University and Genentech, USA
5.	NCT00555672: Study Of Sunitinib In Combination With Cisplatin And 5-Fluorouracil In Patients With Advanced Gastric Cancer. Pfizer, USA
6.	NCT00555620: Study Of Sunitinib In Combination With Cisplatin/Capecitabine Or Oxaliplatin/Capecitabine In Patients With Advanced Gastric Cancer. Pfizer, USA
7.	NCT00553696: Study Of Sunitinib With S-1 And Cisplatin For Gastric Cancer Pfizer, USA
8.	NCT00524186: Sunitinib, Irinotecan, Fluorouracil, and Leucovorin In Treating Patients With Advanced Stomach Cancer or Gastroesophageal Cancer. Roswell Park Cancer Institute and National Cancer Institute (NCI), USA
9.	NCT00450203: Combination Chemotherapy With or Without Bevacizumab in Treating Patients With Previously Untreated Stomach Cancer or Gastroesophageal Junction Cancer That Can Be Removed by Surgery. National Cancer Institute (NCI), USA
10.	NCT00217581: Bevacizumab, Oxaliplatin, and Docetaxel in Treating Patients With Locally Advanced Unresectable or Metastatic Stomach or Gastroesophageal Junction Cancer. Barbara Ann Karmanos Cancer Institute and National Cancer Institute (NCI), USA
11.	NCT00350753: Avastin and Tarceva for Upper Gastrointestinal Cancers. Rigshospitalet, Denmark
12.	NCT00548548: A Study of Bevacizumab in Combination With Capecitabine and Cisplatin as First-Line Therapy in Patients With Advanced Gastric Cancer. Genentech, Hoffmann-La, Roche and Chugai, USA
13.	NCT00178698: Hyperthermia/Thermal Therapy With Chemotherapy to Treat Inoperable or Metastatic Tumors. The University of Texas Health Science Center, Houston, USA
14.	NCT00447330: Xelox (Xeloda + Oxaliplatin) and Avastin for Metastatic Esophagogastric Adenocarcinoma. Duke University, Hoffmann-La Roche, Sanofi-Aventis, and Genentech, USA
15.	NCT00737438: Pre-Operative Chemotherapy Plus Bevacizumab With Early Salvage Therapy Based on PET Assessment of Response in Patients With Locally Advanced But Resectable Gastric and GEJ Adenocarcinoma.Memorial Sloan-Kettering Cancer Center and Genentech, USA
16.	NCT00390416: Study of Docetaxel, Cisplatin, and Fluorouracil (Modified DCF) With Bevacizumab in Patients With Unresectable or Metastatic Gastroesophageal Adenocarcinoma.Memorial Sloan-Kettering Cancer Center, Sanofi-Aventis, Genentech, USA
17.	NCT00172627: Association and Mechanism Between Cyclooxygenase-2 and Interleukin-6 in Gastric Cancer. National Taiwan University Hospital, Taiwan
18.	NCT00565370: Phase I/II XP+Sorafenib in Advanced Gastric Cancer. Asan Medical Center, Asian Stomach cancer Investigator Association will join in the phase II portion, Asia
19.	NCT00595972: Epirubicin Cisplatin and 5-FU Combined With Endostar in Patients With Advanced or Metastatic Gastric Cancer. Fudan University, China
20.	NCT00570531: Phase II Trial for Patients With Loco-Regional Esophageal Carcinoma..University of Michigan Cancer Center and Genentech, USA
21.	NCT00703625: Phase I Study of Docetaxel and Temsirolimus in Resistant Solid Malignancies. Washington University School of Medicine, USA

## FDA-approved antiangiogenic agents in cancer treatment

Anti-angiogenesis studies have led to the development of new agents targeting either angiogenic factors, endothelial cells or other components of the tumor neovasculature (14-22). Many angiogenesis inhibitors have already entered clinical trials in cancer patients.

Anti-angiogenesis is a targeted therapy using drugs or other substances to prevent tumors from creating new blood vessels, so to stop the growth of tumor. Both natural and synthetic anti-angiogenesis inhibitors are being studied, although many of these drugs are still available only in clinical trials, the first anti-angiogenic drug, Bevacizumab (Avastin), was approved by the Food and Drug Administration (FDA) in 2004, for use in the treatment of metastatic colon cancer. Up to now, there have been several angiogenesis inhibitors approved by FDA in cancer treatment (Table 2). These agents, which can interrupt critical cell signaling pathways involved in tumor angiogenesis and growth, consist of three major categories: (a) monoclonal antibodies directed against specific proangiogenic growth factors and/or their receptors; (b) small molecule tyrosine kinase inhibitors (TKIs) of multiple proangiogenic growth factor receptors; and (c) inhibitors of mTOR (mammalian target of rapamycin) represent a smaller category of antiangiogenic therapies with one currently approved agent. In addition, at least two other approved angiogenic agents may indirectly inhibit angiogenesis through mechanisms that are not completely understood.

**Table 2:** FDA-approved angiogenesis inhibitors in oncology

1. Monoclonal antibodies:
Bevacizumab (Avastin), Genentech,
Description: A humanized monoclonal antibody that binds biologically active forms of vascular endothelial growth factor (VEGF) and prevents its interaction with VEGF receptors (VEGFR-1 and VEGFR-2), thereby inhibiting endothelial cell proliferation and angiogenesis.
Approved indications: Metastatic colorectal cancer (mCRC), non-small cell lung cancer (NSCLC), advanced breast cancer.

Cetuximab (Erbitux), Bristol-Myers Squibb, ImClone
Description: A humanized monoclonal antibody that binds biologically active forms of vascular endothelial growth factor (VEGF) and prevents its interaction with VEGF receptors (VEGFR-1 and VEGFR-2), thereby inhibiting endothelial cell proliferation and angiogenesis.
Approved indications: Metastatic colorectal cancer, head and neck cancer.

2. Small molecule tyrosine kinase inhibitors:
Sorafenib (Nexavar), Bayer Onyx
Description: Small molecule TK inhibitor of of VEGFR-1, VEGFR-2, VEGFR-3, PDGFR-ß, and Raf-1.
Approved indications: Advanced renal cell carcinoma, advanced hepatocellular carcinoma.

Sunitinib (Sutent), Pfizer
Description: Small molecule TK inhibitor of VEGFR-1, VEGFR-2, VEGFR-3, PDGFR- ß, and RET.
Approved indications: Advanced renal cell carcinoma, and gastrointestinal stromal tumor (GIST).

3. Inhibitors of mTOR:
Temsirolimus (Torisel™), Wyeth
Description: A small molecule inhibitor of mTOR (mammalian target of rapamycin), part of the PI3 kinase/AKT pathway involved in tumor cell proliferation and angiogenesis.
Approved indications: Advanced renal cell carcinoma.

Other angiogenic inhibitors:
Bortezomib (Velcade®), Millennium,
Description: A proteasome inhibitor that disrupts signaling of the cancer cell, leading to cell death and tumor regression. Bortezomib may have indirect antiangiogenic properties, although the mechanisms are unclear.
Approved indications: Multiple myeloma, mantle cell lymphoma (MCL).

Thalidomide (Thalomid®), Celgene
Description: Possesses immunomodulatory, anti-inflammatory, and antiangiogenic properties, although the precise mechanisms of action are not fully understood.
Approved indications: Multiple myeloma

## Mechanisms of anti-angiogenesis drugs

Anti-angiogenic drugs do not directly attack cancer cells, but target the blood supply necessary for survival and growth of the tumor. In this way, they prevent new tumors formaiton, and cause existing tumors to shrink.

VEGF is one of the most important proteins in tumor angiogenesis. This protein is not made in large amounts by normal cells, but some cancer cells secrete it into the area around them. VEGF then attaches to a VEGF receptor (VEGFR) on the surface of nearby endothelial cells and then switch signals to begin growth and formation of new blood vessels. Many of the anti-angiogenesis drugs used today attack the VEGF pathway. Bevacizumab (Avastin) was the first drug targeted at new blood vessels approved by FDA for use against cancer. This monoclonal antibody is a synthetic version of an immune system protein that binds to VEGF and blocks it from reaching the VEGF receptor (23).

Other drugs, such as sunitinib (Sutent) and sorafenib (Nexavar), are small molecules that attach to the VEGF receptor itself, preventing it from being activated (24, 25). Some other drugs used to treat cancer, such as thalidomide and lenalidomide (Revlimid), are also known to affect blood vessel growth. However, they work against cancer in other ways also (26-30). Drugs that target other blood vessel pathways are now being tested.

Some drugs already used to treat cancer have been found to affect blood vessel growth, too. For example, low dose chemotherapy may prevent tumor growth without causing severe side effects that higher doses would. Some studies suggest the drugs may be effective because they can stop the growth of endothelial cells (28, 31).

## Features of anti-angiogenesis treatment different from traditional chemotherapy

In recent decades, an effort to find drugs without potential severe side effects of chemotherapy has led to the discovery of new drugs that target the tumor itself while sparing normal cells of the body. Traditional chemotherapy drugs work by attacking cells which divide rapidly in the body. Unfortunately, some normal cells, such as those in the mouth mucosa or the digestive tract, also divide rapidly, but they do not differentiate into malignant cells. So these drugs can lead to the side effects including mouth sores, nausea and diarrhea. Anti-angiogenesis drugs do not target normal cells, so in many cases, side effects are milder. However, they may have their own side effects, because many drugs are still being evaluated in the clinical trials, it is not yet known whether side effects will be similar for all drugs in this class.

## Future directions of anti-angiogenesis study

### Finding new anti-angiogenic drugs

More and more new drugs are being developed to target angiogenesis, and some of these new drugs will also target the tumor cells.

Vascular targeting agents (VTAs) are a related group of drugs that may prove to be important in treating cancer (32, 33). Anti-angiogenesis drugs may stop new blood vessels formation, but is there a way to attack tumor blood vessels that have already formed? Some differences have been found between normal blood vessels in the body and those that nourish tumors. Some new drugs may be able to exploit these differences, attacking tumor blood vessels only but sparing normal blood vessels. Several VDAs are now being studied in clinical trials. Early studies have shown that these drugs seem to work best on the inner parts of tumors. This may mean they will work well when combined with other treatments that are more likely to work on the surface of the tumor, such as chemotherapy.

### Combining anti-angiogenesis drugs

It is now clear that tumors can make and release many molecules capable of stimulating angiogenesis. Targeting only one of these chemicals may not be sufficient to induce blockade of tumor angiogenesis, However, combination of drugs that attack different targets may prove to be more effective. Studies on combination of these drugs are under way.

### Combining angiogenesis inhibitors with other therapies

Anti-angiogenesis drugs tend to have milder side effects that are different from other cancer treatments. This makes the idea of combining them with other types of treatment very appealing. Combination of these drugs with chemotherapy drugs, radiation therapy, or other new types of targeted therapies has indicated promising. In the clinical trials, there are currently lots of anti-angiogenesis drugs being studied. Some of these drugs are being tested as single agents, while others are being used in combination with other treatments. Several anti-angiogenesis drugs are now used in the treatment of cancer, and others will be put into use in the near future. Because these are still new drugs, many questions about them remain open. For example, are they most effective used alone or with other treatments? What is the best way of administration? what is the appropriate dosage? These and other important questions are now being studied in clinical trials.

There is some reason to believe that standard chemotherapy drugs and anti-angiogenesis drugs may work well in combination (34). In early clinical trials of bevacizumab (Avastin), it was determined that bevacizumab alone did not help cancer patients survive longer. In subsequent studies, however, it was found that, when combined with other chemotherapy drugs, it was more effective.

### Using metronomic chemotherapy as anti-angiogenesis approach

Most chemotherapy drugs were designed to attack cancer cells directly, but some of them may be useful as anti-angiogenic agents, too. When they are given at low doses over a longer period of time (versus high doses usually given at regular intervals), they seem to work without causing major side effects. This approach is known as metronomic chemotherapy. Some evidences suggest that the chemotherapy, when used this way, may be acting on tumor blood vessels. Several studies are now undergoing to test the value of metronomic chemotherapy, either alone or combined with anti-angiogenic drugs (31, 32).

## Concluding remark

Since so many potent angiogenesis inhibitors are being tested in the clinical trials worldwide, it is optimistic and promising that AIs will be selected as candidates for standard therapy of cancers including gastrointestinal cancers.

## References

[1] Folkman J (1971). Tumor angiogenesis: therapeutic implications. N Engl J Med.

[2] Knighton D, Ausprunk D, Tapper D, Folkman J (1977). Avascular and vascular phases of tumour growth in the chick embryo. Br J Cancer.

[3] Folkman J (1974). Proceedings: Tumor angiogenesis factor. Cancer Res.

[4] Folkman J, Watson K, Ingber D, Hanahan D (1989). Induction of angiogenesis during the transition from hyperplasia to neoplasia. Nature.

[5] Blood CH, Zetter BR (1990). Tumor interactions with the vasculature: angiogenesis and tumor metastasis. Biochim Biophys Acta.

[6] Holmgren L, O'Reilly MS, Folkman J (1995). Dormancy of micrometastases: balanced proliferation and apoptosis in the presence of angiogenesis suppression. Nat Med.

[7] Weidner N, Folkman J, Pozza F, Bevilacqua P, Allred EN, Moore DH, Meli S (1992). Tumor angiogenesis: a new significant and independent prognostic indicator in early-stage breast carcinoma. J Natl Cancer Inst.

[8] Phillips P, Steward JK, Kumar S (1976). Tumour angiogenesis factor (TAF) in human and animal tumours. Int J Cancer.

[9] Harper SJ, Bates DO (2008). VEGF-A splicing: the key to anti-angiogenic therapeutics?. Nat Rev Cancer.

[10] Tait CR, Jones PF (2004). Angiopoietins in tumours: the angiogenic switch. J Pathol.

[11] Roccaro AM, Vacca A, Ribatti D (2006). Bortezomib in the treatment of cancer. Recent Patents Anticancer Drug Discov.

[12] Yoshikawa T, Yanoma S, Tsuburaya A, Kobayashi O, Sairenji M, Motohashi H, Noguchi Y (2000). Angiogenesis inhibitor, TNP-470, suppresses growth of peritoneal disseminating foci. Hepatogastroenterology.

[13] Shah MA, Ramanathan RK, Ilson DH, Levnor A, D'Adamo D, O'Reilly E, Tse A (2006). Multicenter phase II study of irinotecan, cisplatin, and bevacizumab in patients with metastatic gastric or gastroesophageal junction adenocarcinoma. J Clin Oncol.

[14] Fotsis T, Zhang Y, Pepper MS, Adlercreutz H, Montesano R, Nawroth PP, Schweigerer L (1994). The endogenous oestrogen metabolite 2-methoxyoestradiol inhibits angiogenesis and suppresses tumour growth. Nature.

[15] Brouty-Boye D, Zetter BR (1980). Inhibition of cell motility by interferon. Science.

[16] Taylor S, Folkman J (1982). Protamine is an inhibitor of angiogenesis. Nature.

[17] Folkman J, Langer R, Linhardt RJ, Haudenschild C, Taylor S (1983). Angiogenesis inhibition and tumor regression caused by heparin or a heparin fragment in the presence of cortisone. Science.

[18] Ingber D, Fujita T, Kishimoto S, Sudo K, Kanamaru T, Brem H, Folkman J (1990). Synthetic analogues of fumagillin that inhibit angiogenesis and suppress tumour growth. Nature.

[19] Rastinejad F, Polverini PJ, Bouck NP (1989). Regulation of the activity of a new inhibitor of angiogenesis by a cancer suppressor gene. Cell.

[20] O'Reilly MS, Holmgren L, Shing Y, Chen C, Rosenthal RA, Moses M, Lane WS (1994). Angiostatin: a novel angiogenesis inhibitor that mediates the suppression of metastases by a Lewis lung carcinoma. Cell.

[21] O'Reilly MS, Boehm T, Shing Y, Fukai N, Vasios G, Lane WS, Flynn E (1997). Endostatin: an endogenous inhibitor of angiogenesis and tumor growth. Cell.

[22] D'Amato RJ, Loughnan MS, Flynn E, Folkman J (1994). Thalidomide is an inhibitor of angiogenesis. Proc Natl Acad Sci U S A.

[23] Ng K, Meyerhardt JA, Fuchs CS (2007). Adjuvant and neoadjuvant approaches in gastric cancer. Cancer J.

[24] Susman E (2006). New drug increases survival in stomach cancer. Lancet Oncol.

[25] Gold JS, Dematteo RP (2006). Combined surgical and molecular therapy: the gastrointestinal stromal tumor model. Ann Surg.

[26] Pro B, Younes A, Albitar M, Dang NH, Samaniego F, Romaguera J, McLaughlin P (2004). Thalidomide for patients with recurrent lymphoma. Cancer.

[27] Rossi A, Gridelli C, Bria E (2008). Thalidomide in small-cell lung cancer: is it a tombstone?. J Clin Oncol.

[28] Blansfield JA, Caragacianu D, Alexander HR, Tangrea MA, Morita SY, Lorang D, Schafer P (2008). Combining agents that target the tumor microenvironment improves the efficacy of anticancer therapy. Clin Cancer Res.

[29] Galustian C, Meyer B, Labarthe MC, Dredge K, Klaschka D, Henry J, Todryk S (2008). The anti-cancer agents lenalidomide and pomalidomide inhibit the proliferation and function of T regulatory cells. Cancer Immunol Immunother.

[30] Lu L, Payvandi F, Wu L, Zhang LH, Hariri RJ, Man HW, Chen RS (2008). The anti-cancer drug lenalidomide inhibits angiogenesis and metastasis via multiple inhibitory effects on endothelial cell function in normoxic and hypoxic conditions. Microvasc Res.

[31] Munoz R, Shaked Y, Bertolini F, Emmenegger U, Man S, Kerbel RS (2005). Anti-angiogenic treatment of breast cancer using metronomic low-dose chemotherapy. Breast.

[32] Huxham LA, Kyle AH, Baker JH, McNicol KL, Minchinton AI (2008). Exploring vascular dysfunction caused by tirapazamine. Microvasc Res.

[33] Thorpe PE (2004). Vascular targeting agents as cancer therapeutics. Clin Cancer Res.

[34] Browder T, Butterfield CE, Kraling BM, Shi B, Marshall B, O'Reilly MS, Folkman J (2000). Antiangiogenic scheduling of chemotherapy improves efficacy against experimental drug-resistant cancer. Cancer Res.

